# Lead Poisoning

**Published:** 1852-10

**Authors:** 


*Lead Poisoning.* Prof. Frost, of the Medical College of S. C., reports in the September number of the *Charleston Medical Journal*, several cases of *lead cholic* and other forms of *lead poisoning*, which, in his opinion, were clearly traceable to the use of water impregnated with some salt of lead, either taken from soda water reservoirs or from leaden pipes in other ways. As regards the asserted deleterious operation of acetate of lead in the ordinary medicinal doses (to which Dr. Jaynes, of Virginia, and others have lately called attention in the journals,) Dr. Frost avers that "he has never, in the course of his practice, known any injurious effects to arise from the use of this article."

